# A Study on the Impact of Poly(3-hexylthiophene) Chain Length and Other Applied Side-Chains on the NO_2_ Sensing Properties of Conducting Graft Copolymers

**DOI:** 10.3390/s18030928

**Published:** 2018-03-20

**Authors:** Marcin Procek, Kinga Kepska, Agnieszka Stolarczyk

**Affiliations:** 1 Department of Optoelectronics, Silesian University of Technology, 2 Krzywoustego Street, 44-100 Gliwice, Poland; 2 Department of Physical Chemistry and Technology of Polymers, Silesian University of Technology, 9 Strzody Street, 44-100 Gliwice, Poland; Kinga.Kepska@polsl.pl (K.K.); Agnieszka.Stolarczyk@polsl.pl (A.S.)

**Keywords:** gas sensor, NO_2_ sensor, P3HT, graft copolymers, tailor-made receptor materials, low temperature gas sensing, functionalized conducting polymers

## Abstract

The detection and concentration measurements of low concentrations of nitrogen dioxide (NO_2_) are important because of its negative effects on human health and its application in many fields of industry and safety systems. In our approach, conducting graft copolymers based on the poly(3-hexylthiophene) (P3HT) conducting polymer and other side-chains, polyethylene glycol (PEG) and dodec-1-en, grafted on a poly(methylhydrosiloxane) backbone, were investigated. The grafts containing PEG (PEGSil) and dodec-1-en (DodecSil) in two variants, namely, fractions with shorter (hexane fraction -H) and longer (chloroform fraction -CH) side-chains of P3HT, were tested as receptor structures in NO_2_ gas sensors. Their responses to NO_2_, within the concentration range of 1–20 ppm, were investigated in an nitrogen atmosphere at different operating temperatures—room temperature (RT) = 25 °C, 50 °C, and 100 °C. The results indicated that both of the copolymers with PEG side-chains had higher responses to NO_2_ than the materials with dodec-1-en side-chains. Furthermore, the results indicated that, in both cases, H fractions were more sensitive than CH fractions. The highest response to 1 ppm of NO_2,_ from the investigated graft copolymers, had PEGSil H, which indicated a response of 1330% at RT and 1980% at 100 °C. The calculated lower-limit of the detection of this material is lower than 300 ppb of NO_2_ at 100 °C. This research indicated that graft copolymers of P3HT had great potential for low temperature NO_2_ sensing, and that the proper choice of other side-chains in graft copolymers can improve their gas sensing properties.

## 1. Introduction

In the contemporary era of civilization, one of the major problems facing society is air pollution. One of the most common forms of pollution is nitrogen dioxide (NO_2_), which is associated with adverse effects on human health. This is because, at high concentrations, it can cause inflammation of the airways [1]. NO_2_ also contributes to the formation of secondary particulate aerosols and tropospheric ozone (O_3_) in the atmosphere—both are significant air pollutants due to their adverse effects on human health [2]. Thus, NO_2_ monitoring is important in the automotive and the energetic industries [3,4]. The nitro compounds are also components of vapors of explosive materials, such as trinitrotoluene, Research Department Explosive, and nitroglycerine [5,6,7].

The most popular gas sensors that are used in NO_2_ concentration monitoring are chemoresistive gas sensors that are based on metal oxide (MOX) semiconducting receptors, such as SnO_2_, TiO_2_, ZnO, and others [8,9,10,11]. These gas sensors have many advantages, such as high sensitivity and long-term stability. However, in contrast, these materials exhibit poor selectivity and operate at relatively high temperatures (often higher than 200 °C) [12,13].

Conducting polymers (CPs) have been studied as alternative receptor materials for gas sensing since the early 1980s. Polymers—such as polyaniline, polypyrrole, polythiophene (PTh), their derivatives, and others—were studied extensively with regards to their use in gas sensors [14,15]. These materials showed some advantages, such as high sensitivity, short response times, and low operation temperatures, often room temperature (RT) [14,16]. The polymer gas sensors showed good sensing properties for NH_3_, NO_2_, I_2_, H_2_S, and volatile organic compounds (VOCs) [14,17]. CPs have been successfully applied in many types of chemical gas sensors, such as electrical (resistance, thin film transistors), mass sensitive (on surface acoustic wave and quartz crystal microbalance transducers), optical (based on surface plasmon resonance, optical fibers, fluorescence, etc.), and others [14,17,18,19,20].

Unfortunately, CPs that are in their pristine and doped form are often not able to be processed (insoluble and infusible), making such materials unattractive for mass production. For PTh, this problem was solved through functionalization, by the addition of an alkyl side-chain. These materials are named poly(alkylthiophenes) (PATs), and are soluble in many common solvents, such as chloroform, hexane, chlorobenzene, etc., making them convenient for further processing [21].

The intrinsically conducting polymers are π-conjugated macromolecules that show electrical and optical changes in property when they are doped/dedoped by some chemical agents. Among the π-conjugated polymers, poly(3-hexylthiophenes) (P3HTs) have gained particular interest because of their high mobility of charge carriers, good stability, the ability to process from a solution, and the ability to self-organize. These features are, however, the result of a number of conditions, the most important of these being the length of the polymer chain and its topology [21,22].

The major problem with the CPs applied in sensors is their fast aging processes, which make them chemically and electrically unstable in the long term [23]. Furthermore, PATs, especially regioregular (rr) ones, have poor mechanical and adhesive properties, making them more susceptible to damage [24].

The literature describes many proposed methods for improving the electrical, mechanical, and gas sensing properties of CPs. These methods include blending with other polymers, hybridization with inorganic materials (such as MOX, graphene oxide etc.), chemical modification of the polymer chain, copolymerization, and grafting, among others [17,23,25,26,27,28].

Within the scope of this paper, the objective is to develop multi-functional, π-conjugated materials designed to meet the demands of sensor fabrication. Observing the potential of the organic devices, we synthesized the graft copolymers of siloxane and π-conjugated macromolecules of rrP3HT. These materials, featuring various electron affinities, can be tailored on both a molecular and a supramolecular level, in order to exhibit a range of physicochemical properties. Therefore, our idea for a copolymer—featuring numerous degrees of freedom according to the design of its architecture—coupled with the specific properties of its constituents, will result in a new class of sensing materials.

In this paper, we report the influence of the π-conjugated polymer, namely the P3HT chain length, and other comonomers, such as polyelectrolyte-polyethylene glycol (PEG) and the internal plasticiser (dodec-1-en), on the NO_2_ sensing properties of grafted comb-like copolymers with a poly(methylsiloxane) backbone. The two graft copolymers, PEGSil (Poly(dimethylsiloxane)-co-[poly(metylhydrosiloxane)-graft-2-vinyl-poly(3-hexylthiophene)]-co-[poly(metylhydrosiloxane)-graft-poly(ethylene glycol) methyl ether methacrylate)]) and DodecSil (Poly(dimethylsiloxane)-co-[poly(metylhydrosiloxane)-graft-2-vinyl-poly(3-hexylthiophene)]-co-[poly(metylhydrosiloxane)-graft-dodec-1-en]), with different P3HT chain lengths, were tested as gas receptor thin films in resistance NO_2_ sensors.

This paper presents material synthesis and characterization, a sensors fabrication process description, and a study of the NO_2_ sensing properties of the obtained devices. Our research shows that the proper choice of CP chain length and other side-chains in graft copolymers can improve their gas sensing properties. The investigated materials show higher responses to single ppms of NO_2_ than they do to other P3HT-based materials that are functionalized by other methods. Thus, we conclude that the grafting method appears to be a prospective way of obtaining tailor-made gas sensing materials.

## 2. Materials and Methods

**Figure 1:**
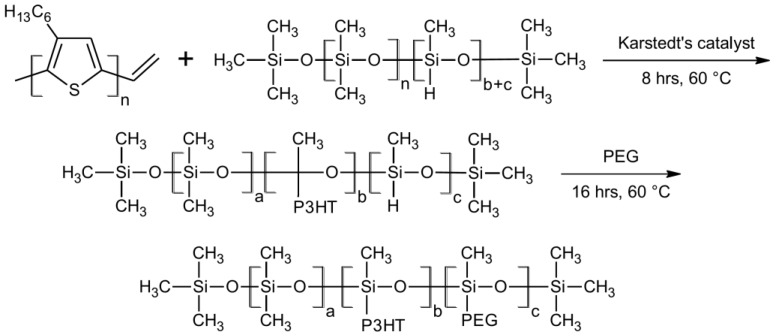
Scheme of the poly(3-hexylthiophenes) (P3HT) graft copolymer synthesis route.

The synthesis of the vinyl terminated regioregular poly(3-hexylthiophene) (vin-rrP3HT), via the Grignard Metathesis Method (GRIM) method, and the synthesis of the vinyl terminated rrP3HT, was conducted based on the procedure described in the literature in [29] (all of the materials were from Sigma Aldrich, Saint Louis, MO, USA). The polymers of two average M_n_, namely, a 10,000-chloroform fraction (CH) and a 4000-hexane fraction (H)—as determined by the gel permeation chromatography (GPC) calibrated on the polystyrene standard—were prepared. The calculated average of the P3HT repeat units were 60 and 24 for the CH and H fractions, respectively. The investigated graft polymers were obtained using the method described in the patent application [30]. The synthesis was based on the grafting of the vinyl terminated P3HT with a different chain length and PEG, or dodec-1-en onto poly(methylhydrosiloxane) (PMHS) chains (all of the materials were from Sigma Aldrich, Saint Louis, MO, USA). The scheme of the synthesis of the obtained P3HT graft copolymers is presented in Figure 1.

The chemical structures of the investigated materials were confirmed by ^1^H Nuclear Molecular Resonance (^1^H-NMR) and Fourier-transform infrared spectroscopy with the attenuated total reflectance (FTIR-Atr). The IR spectroscopy was carried out on a Perkin-Elmer Spectrum-Two (Waltham, MA, USA) spectrometer with a Universal Attenuated Total Reflectance accessory UATR (Single Reflection Diamond) module.

An ^1^H-NMR analysis of products was performed for solutions in CDCl_3_ on a Varian Unity Inova (Palo Alto, CA, USA) spectrometer with a resonance frequency of 300 MHz, using tetramethylsilane (TMS) as the internal standard.

The obtained graft copolymers and polymers, which are the receptor materials, were deposited on the interdigital transducers (IDT) using spin-coating method. The IDTs with gold electrodes on the Si/SiO_2_ substrates were described in detail in our previous works [11,18]. All of the polymers were dissolved in chloroform (CHCl_3_) (POCH, Gliwice, Poland) in the proportion of 2.5 mg of polymer to 1 mL of solvent. The solutions were dropped on the rotating transducers in amounts of 25 μL. During all of the coating processes, the spin rate of the IDTs were kept on a constant level of 500 rpm. Further details about the spin-coating process can be found in our previous work [18], where different graft copolymers and pure P3HT that were obtained by the same technological process, were tested as NO_2_ receptor materials.

The obtained sensing films were characterized using the atomic force microscopy (AFM) technique using the NTGRA Prima system (NT-MDT, Moscow, Russia) with a semi-contact mode and using HA-HR probes (NT-MDT) with a 260 kHz work frequency. The typical curative radius of the tip of the used probes is less than 10 nm, the tip length is ≤1 μm, and the cantilever length is 123 μm. All of the measurements were performed using a scanning frequency of 0.5 Hz (scan speed for 5 × 5 μm areas was approximately 5 μm/s). The AFM data were processed and analyzed using the dedicated software Nova 1.1.0.1824 (NT-MDT, Moscow, Russia).

The sensors were placed on a thick film heater on an alumina substrate and were electrically connected with chip feedthroughs using an ultrasonic wire bonding method with 25 μm of gold wire. The temperature was controlled using a SR94 controller (Shimaden, Tokyo, Japan) with a Pt100 temperature sensor. Further details concerning the sensors’ electrical connections, heating, and temperature control can be found in our previous work [11]. The four sensors (each with a different graft copolymer) were tested simultaneously using a testing chamber and mass flow controllers that were based on a measurement system that was also presented in detail in our previous work [11]. The sensors’ resistance was measured using a multi-switch unit 34970A (Agilent, Santa Clara, CA, USA) within a 10 MΩ range.

The sensors’ reaction to the small concentrations of NO_2_ within the range of 1 ppm to 20 ppm, in different operating temperatures (RT = 25 °C, 50 °C, and 100 °C), were tested. The gas mixture was prepared from a calibration mixture (100 ppm NO_2_ in N_2_) and a carrier gas (pure N_2_), using gas a dosing system that was based on the mass flow controllers. The research was conducted in a nitrogen atmosphere in order to describe the interactions of the polymeric materials with NO_2,_ without the interference of oxygen and other reactive gases. During all of the experiments, the relative humidity (RH) of the gas mixture was kept at a constant level of 6 ± 1%.

The sensors’ responses were calculated according to the following formula:

(1) $$\mathrm{Response}=\frac{R_{a}}{R_{g}},$$

where *R_a_* is a base resistance in a pure carrier gas and *R_g_* is a resistance in a target gas.

## 3. Results and Discussion

### 3.1. Materials Characterization

**Figure A1:**
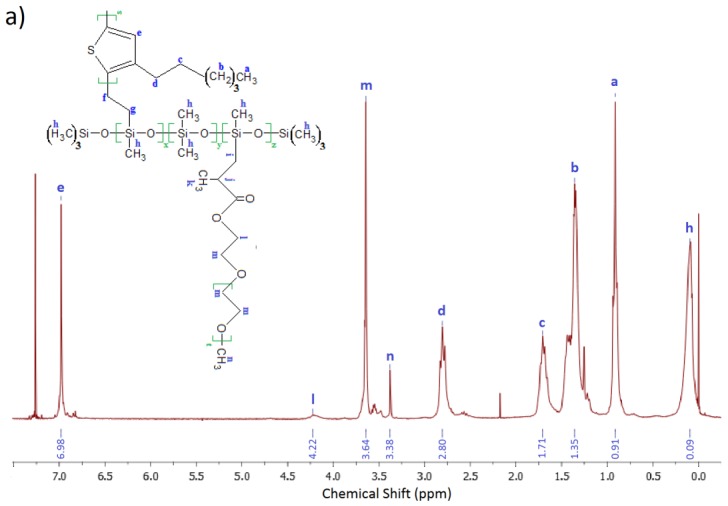

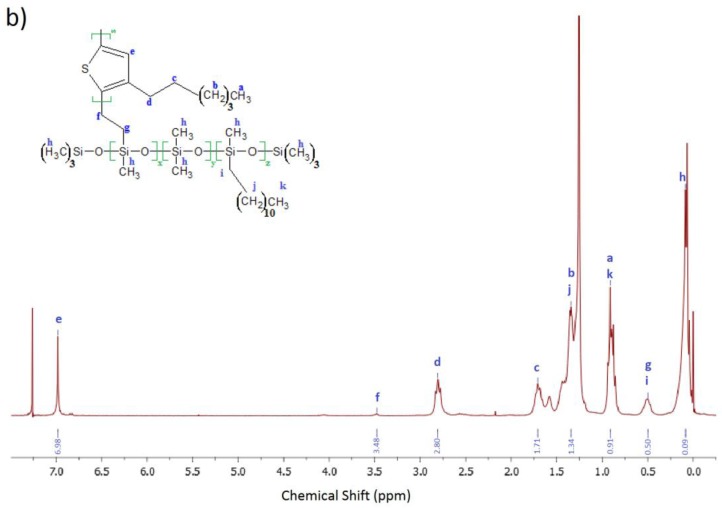
^1^H Nuclear Molecular Resonance (^1^H-NMR) spectra of (**a**) PEGSil CH; (**b**) DodecSil CH. Marked by letters of the hydrogen atoms group assigned to particular observed signal groups visible in the ^1^H-NMR spectrum. Polymers’ macroparticles schemes are shown in the spectrograms.

Both FTIR and ^1^H-NMR were used in order to identify and characterize the four copolymers. The assignments of the chemical shifts are presented in Table 1. The NMR has allowed for the compositional and structural determination of each copolymer. The ^1^H-NMR spectra of the copolymer DodecSil CH and PEGSil CH, presented in Figure A1 (Appendix), show the representative spectra for all of the copolymers that were used used (both with P3HT chain lengths).

**Figure A2:**
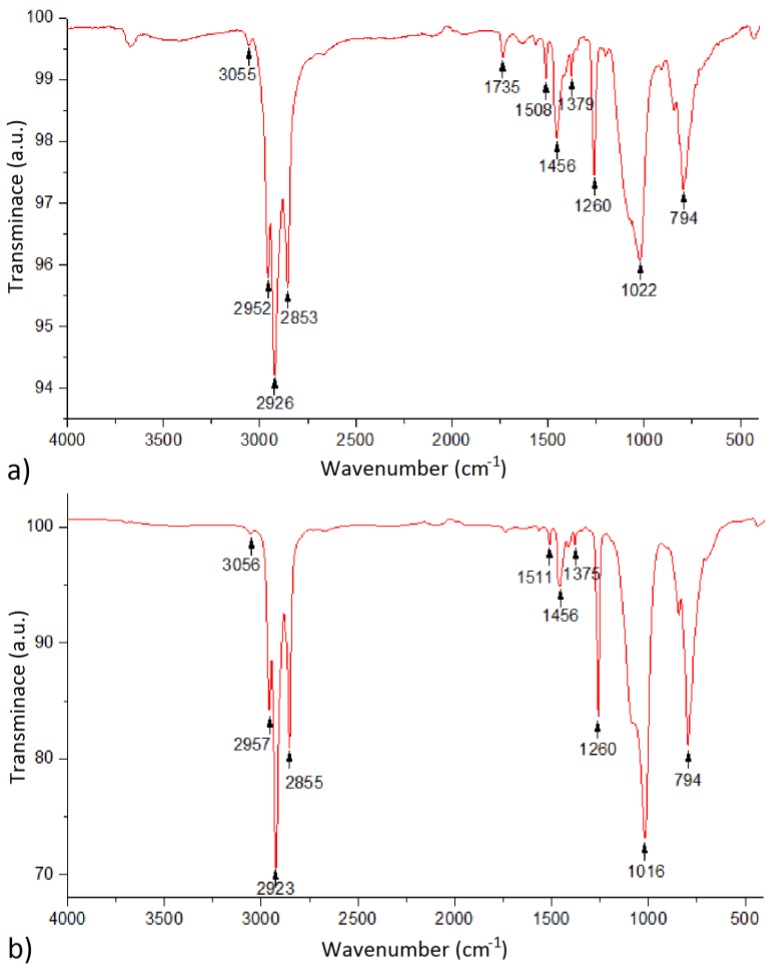
Fourier-transform infrared spectroscopy with the attenuated total reflectance (FTIR-Atr) spectra of (**a**) PEGSil CH; (**b**) DodecSil CH.

In Figure A2, the FTIR-Atr representative spectra of graft copolymers are presented. Detailed analyses of the IR signals are presented in Table 2. The spectra of the grafted copolymers, not only shows all of the characteristic peaks of the siloxane groups of Si-O-Si at 1090 cm^−1^ and at 1020 cm^−1^ and those of P3HT, but also shows the absorption bands of poly(ethylene glycol), such as CH_2_ and CH_3_, bending at 1467.8 cm^−1^ and at 1342.4 cm^−1^ C–O–C. This indicates that the P3HT, poly(ethylene glycol), and dodecyl were successfully grafted onto the polysiloxane backbone.

**Figure 2:**
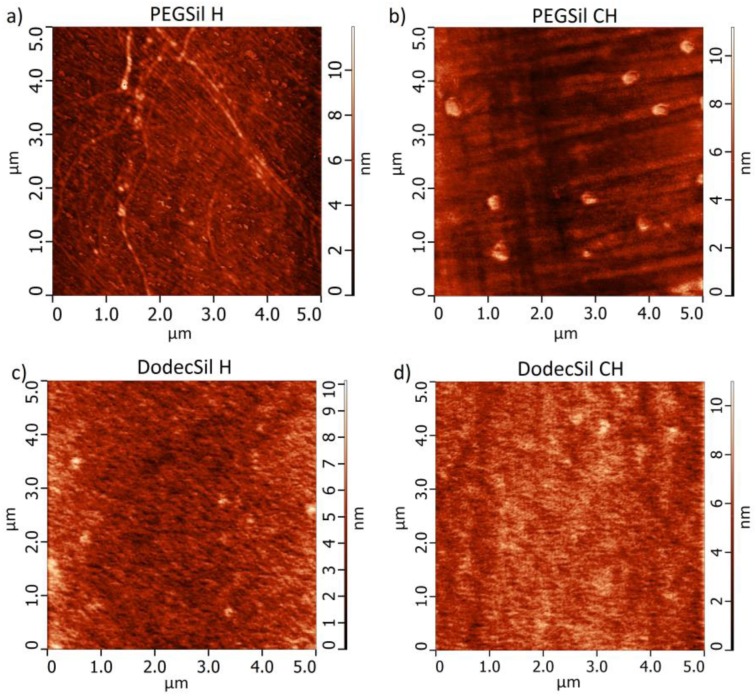
Atomic force microscopy (AFM) height images from 5 × 5 μm areas between the electrodes of (**a**) PEGSil H; (**b**) PEGSil CH; (**c**) DodecSil H; (**d**) DodecSilCH.

**Figure A3:**
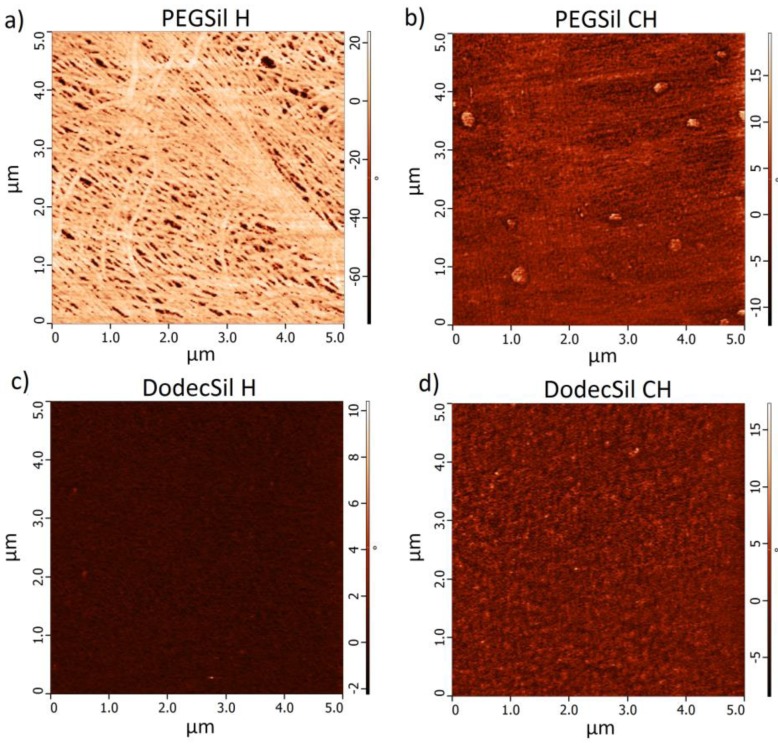
Atomic force microscopy (AFM) phase images from 5 × 5 μm areas between the electrodes of (**a**)PEGSil H; (**b**) PEGSil CH; (**c**) DodecSil H; (**d**) DodecSilCH.

The morphology of all of the prepared polymer sensing films was investigated using the AFM method. These measurements showed that all of the films were homogenous and smooth, with root mean squares (RMS) smaller than 2 nm (Table 3). Since all of the results were similar, the representative AFM height of the images (of 5 × 5 μm areas between the IDT electrodes) has been presented in Figure 2 (the complementary phase images are presented in Figure A3). The thickness of all of the films were measured using the AFM method and found to be approximately 20 ± 5 nm. The base resistance of the sensing structures measured at RT in pure N_2_ are presented in Table 3.

### 3.2. Gas Sensing Mesurements

The conductivity of the studied graft copolymers is provided by the presence of the CP (P3HT) chains. The application of the polymethylsiloxane backbone is responsible for the positive film forming process and adhesive properties. Additionally, siloxane has good gas permeability values and, as a result it does not significantly interfere with the gas diffusion processes in sensing films. The other side-chains, PEG or dodec-1-en, were applied to improve the mechanical properties of the investigated graft copolymers. PEG is a well-known polyelectrolyte, which increases gas and charge transfer in the sensing films. PEG has positive NO_x_ adsorption properties [31]. Thus, it is added to our grafts in order to improve the material sensing properties, while dodec-1-en is only the internal plasticizer and should not show additional interaction with the analyte. Thus, the addition of this comonomer will improve the mechanical properties of the sensing material.

In all cases, NO_2_ causes a decrease of the sensors’ resistance (this is a response increase) as it is a typical p-type semiconductor reaction to the oxidizing gas action. The sensing mechanism is based on the P3HT doping process, where NO_2_ is an electron acceptor dopant [32,33]. The reaction of P3HT with NO_2_ leads to an increase in the holes of concentration, because of the formation of polarons followed by bipolaron formation [22,34].

**Figure 3:**
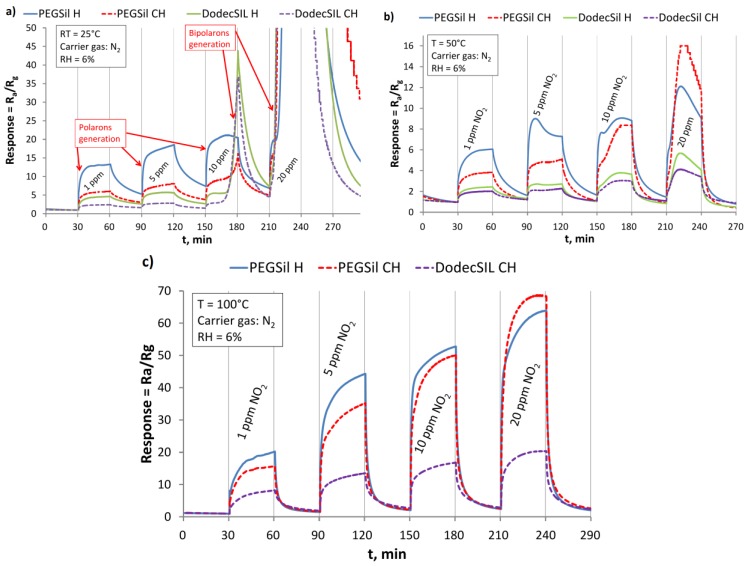
Sensor responses to different nitrogen dioxide (NO_2)_ concentrations at (**a**) RT = 25 °C; (**b**) 50 °C; (**c**) 100 °C.

For low concentrations of NO_2_ (lower than 10 ppm) at RT, polarons are generated, causing the resistance to drop, seen in Figure 3a. However, all of the investigated materials stay in their semiconducting states. After some time at higher NO_2_ concentrations, the polaron concentration becomes sufficient for bipolaron generation to occur. This can be seen in Figure 3a, for 10 and 20 ppm peaks, where a very fast resistance drop (response increase) to small values (even hundreds of ohms) is observed. Consequently, polymers switched to conducting states. Furthermore, the differences between the time and concentration of where bipolaron generation starts shows that CP chain length has an influence on this process [35,36]. P3HT H has a shorter effective conjugation length and prefers to generate stable polarons, in contrast to longer P3HT CH chain, where the generation of bipolarons is more probable. This leads us to conclude that, for NO_2_ sensing applications, the semiconducting state of P3HT is required for proper NO_2_ concentration measurement, and its conducting state is an upper limit for the proper operation sensors. However, it has to be stressed that the adsorption processes are reversible in all cases, after the NO_2_ removal sensor resistance returns to its initial value. Unfortunately, at RT, the regeneration process is slow (Figure 3a) and an operating temperature elevation is required to improve sensor dynamics.

At higher temperatures (50 °C and 100 °C), a switch to the conducting state was not observed in measured the concentration range, as seen in Figure 3b,c. Thermal activation causes faster NO_2_ desorption/polarons recombination. Consequently the bipolarons were not generated, the sensor operated faster, and regeneration was more efficient.

**Figure 4:**
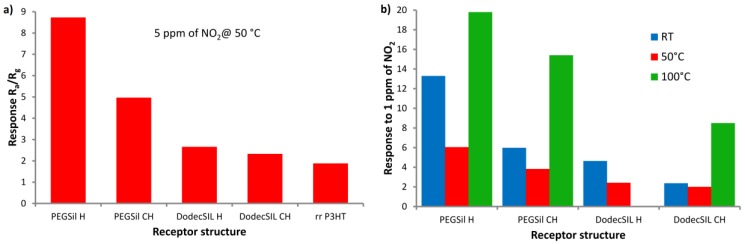
Summary of sensor responses to (**a**) 5 ppm of NO_2_ at 50 °C, in reference to rrP3HT response taken from our previous work [18]; (**b**) 1 ppm of NO_2_ at different temperatures.

The significant differences in sensor responses were observed between graft copolymers with PEG and those with dodec-1-en side-chains. According to our prediction, PEG improved sensor responses due to the abovementioned interaction with NO_2_. The sensor responses to 5 ppm of NO_2_ at 50 °C, shown in Figure 4a, and to 1 ppm of NO_2_ at different temperatures, shown in Figure 4b, are collected and shown in Figure 4 for an easy comparison. It is evident that, in all cases, PEGSils’ responses were higher than those of DodecSils. Furthermore, in all cases, H fractions exhibited higher responses to NO_2_ than CH fractions did. The highest responses were observed at the temperature of 100 °C. In this case, the sensors showed the best stability and operation dynamics out of all the used operating temperatures.

**Figure 5:**
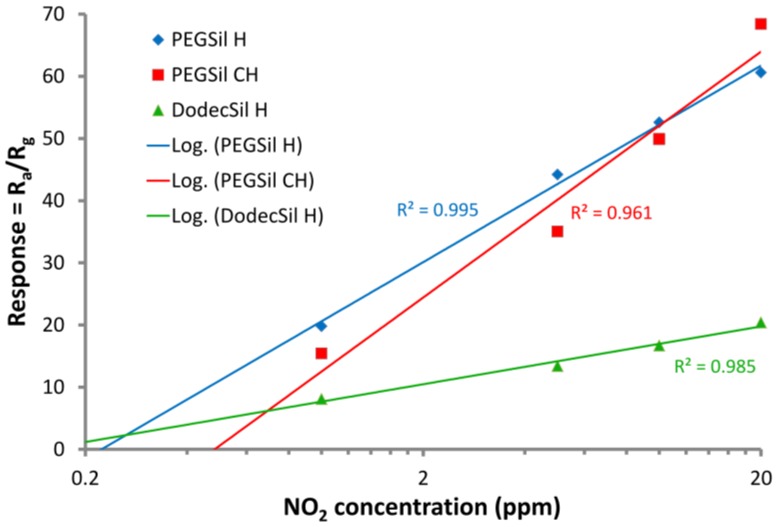
Sensors’ calibration curves in a semi-logarithmic scale for an operating temperature = 100 °C.

The sensors’ scalability at 100 °C was confirmed using the calibration curves presented in Figure 5. In this case, the gas sensors demonstrate the logarithmic dependence between the response and the concentration. In order to show the sensors’ potential for low concentration detection, the calibration curves are presented in a semi-logarithmic scale with logarithmic approximations. Figure 5 shows that the proposed sensors have great potential for sub-ppm and ppb levels of NO_2_ concentration measurements.

Table 4 presents a comparison of the responses to NO_2_ of PEGSil H and other P3HT-based receptor materials that were recently investigated in the literature. This summary shows that the graft copolymers investigated in our current and previous work [18] show higher responses to NO_2_ than clean P3HT and P3HT that are functionalized using other methods. In this case, the responses to 1 ppm of NO_2,_ at all measured temperatures were significantly higher than the responses of the other compared materials’ responses to 4 ppm or 5 ppm of NO_2_ (Table 4). This confirms that the investigated materials have a greater potential for the measuring of NO_2_ in sub-ppm and ppb concentration levels than other P3HT-based materials. Thus, we can conclude that our strategy for improving the NO_2_ sensing properties of CPs, using the grafting method, is a promising advance in the research of tailor-made functionalized receptor materials. The obtained results show that the responses of the proposed graft copolymers are comparable with MOX based sensors and can be considered as an alternative to these sensors for NO_2_ sensing. The advantages of these materials include a relatively low operating temperature and the numerous technologies available for the application on transducers, (such as spin-coating, printing, drop-coating, and dip-coating methods). However, long-term stability and selectivity of such materials still require further investigation.

## 4. Conclusions

In summary, this work presents the results of research on new graft copolymers that contain the semiconductor rrP3HT chain in their structures. The aim of the work was to examine the sensor’s response to NO_2,_ in order to check whether the addition of rrP3HT to the polymethylhydrosiloxane chain, and the additional grafting of various types of comonomers, would affect the rrP3HT sensor properties. Our approach shows that the proper selection of the graft copolymers, and the length of CP chains, improve the sensing properties of the polymer gas receptor material. The investigated materials show higher responses to single ppms of NO_2_ than other P3HT-based materials (cleaned or functionalized by other methods). These responses are comparable to many MOX-based sensors and can be considered as an alternative to these sensors for NO_2_ sensing. The advantages of the proposed grafted copolymers-based sensors include their ability to operate at a relatively low temperature and the numerous technologies available for the application of such materials on transducers. The obtained sensing structures show the potential to measure low concentrations at sub-ppm and ppb level, making them useful for many applications. Based on these results found in our research, we presented a novel alternative method of obtaining the tailor-made gas sensing materials, based on CPs.

## Figures and Tables

**Table 1:** Assignments of the ^1^H-Nuclear Molecular Resonance (NMR) of DodecSil CH and PEGSil CH signals.

Sample	^1^H-NMR δ (ppm)
DodecSil CH	h: 0,09 (C**H**_3_-Si-O-); g, i: 0,50 (−C**H**_2_-Si-O-) a, k: 0,91 (−CH_2_-C**H**_3_); b, j: 1,34 (−(C**H**_2_)_3_-); c: 1,71 (−C**H**_2_-CH_2_-C_Ar_); d: 2,80 (−C**H**_2_-C_Ar_); f: 3,48 (−Si-CH_2_-C**H**_2_-C_Ar_); e: 6,98 (**H**-C_Ar_)
PEGSil CH	h: 0,09 (C**H**_3_-Si-O-); a: 0,91 (−CH_2_-C**H**_3_); b: 1,36 (−(C**H**_2_)_3_-); c: 1,71 (−C**H**_2_-CH_2_-C_Ar_); d: 2,80 (−C**H**_2_-C_Ar_); n: 3,38 (C**H**_3_-O-); m: 3,64 (−C**H**_2_-O-); l: 4,22 (CH_2_-C**H**_2_-C(=O)-); e: 6,98 (**H**-C_Ar_)

**Table 2:** Assignments of the main Fourier-transform infrared spectroscopy with attenuated total reflectance (FTIR-Atr) features of DodecSil CH and PEGSil CH.

Sample	ν (cm^−1^) Assignment
DodecSil CH	794 [δ^1^(**Si-(CH_3_)_2_**), s^2^]; 1016 [ν_asym._(**Si-O-Si**), vs]; 1260 [δ_asym._(**Si-CH_3_**), s]; 1375 [δ_sym._(**-CH_2_**-), w]; 1456 [ν_asym_(**C_Ar_ = C_Ar_**), m]; 1511 [ν_sym_(**C_Ar_ = C_Ar_**), w]; 2855 [ν_sym._(**C-H**), s]; 2923 [ν_asym._(**C-H**), vs]; 2957 [ν_sym._(**C-H**), s]; 3056 [ν(**C_Ar_-H**), w]
PEGSil CH	795 [δ(**Si-(CH_3_)_2_**), s]; 1022 [ν_asym._(**Si-O-Si**), vs]; 1260 [δ_asym._(**Si-CH_3_**), s]; 1379 [δ_sym._(**-CH_2_**-), w]; 1456 [ν_asym_(**C_Ar_ = C_Ar_**), m]; 1508 [ν_sym_(**C_Ar_ = C_Ar_**), w]; 1735 [ν(**C = O**), w]; 2853 [ν_sym._(**C-H**), s]; 2926 [ν_asym._(**C-H**), vs]; 2952 [ν_sym._(**C-H**), s]; 3055 [ν(**C_Ar_-H**), w]

^1^ vibration type: ν—stretching; δ—deformation; sym.—symetric; asym.—asymetric; ^2^ band intensity: w—weak; m—medium; s—strong; vs—very strong.

**Table 3:** Root mean square (RMS) roughness values calculated from 5 × 5 μm areas between the electrodes and base resistance at room temperature (RT) in N_2_ for all sensing structures.

Sample	PEGSil H	PEGSil CH	DodecSil H	DodecSil CH
RMS (5 × 5 μm), nm	1.24 ± 0.20	1.21 ± 0.20	1.05 ± 0.10	1.06 ± 0.10
Base Resistance, kΩ	1087.5 ± 1.0	128.7 ± 1.0	18496.4 ± 1.0	113.3 ± 1.0

**Table 4:** Comparison of the responses of P3HT-based receptor structures to NO_2_.

Material	NO_2_ Concentration	Response	Operating Temperature	Reference
PEGSil H	1 ppm	1330% 605% 1980%	RT 50 °C 100 °C	Current work
rrP3HT	5 ppm	188%	50 °C	[18]
P3HT	5 ppm	11% *	RT	[33]
Poly(methylsiloxane)-graft-poly(3-hexylthiophe)-graft-poly(ethylene glycol)	5 ppm	2300%	50 °C	[18]
(P3HT) ZnO@GO hybrid	5 ppm	210%	50 °C	[37]
P3HT/ZnO NS-NR composite	4 ppm	60% *	RT	[38]
RGO-P3HT composite	4 ppm	40% *	RT	[39]
P3HT:ZnO (ratio6:2) hybrid	5 ppm	20% *	RT	[40]
P3HT-SnO_2_composite	5 ppm	500%	100 °C	[41]
Poly-(bisdodecylquaterthiophene)	5 ppm	300% *	RT	[26]
poly-(bisdodecylthioquaterthiophene)	5 ppm	450% *	RT	[26]
P3HT:ZnO-nanowire composite	4 ppm	30% *	RT	[42]

* Response calculated as (R_a_ − R_g_)/R_a_ or (I_a_ − I_g_)/I_a_.
